# Tau protein differentially affects Piezo1 and Kir2.1 channels in brain capillary endothelial cells

**DOI:** 10.1016/j.bpj.2026.07.003

**Published:** 2026-07-03

**Authors:** Trevor Harriman, Osama F. Harraz

**Affiliations:** 1 Department of Pharmacology, Larner College of Medicine, Vermont Center for Cardiovascular and Brain Health, University of Vermont, Burlington, VT, USA

## Abstract

Accumulation of amyloid-β (Aβ) peptides and Tau proteins in the brain is a hallmark of neurodegeneration. Such build-up forms Aβ plaques and Tau neurofibrillary tangles, both of which are associated with synaptic loss, cognitive decline, and reduced cerebral blood flow in Alzheimer’s disease (AD). Two ion channels in brain capillary endothelial cells (ECs)—the inwardly rectifying potassium channel Kir2.1 and the mechanosensitive channel Piezo1—are critical regulators of cerebral blood flow, and both display impaired activity in AD. Whether Aβ and Tau affect these channels remains incompletely understood. Using patch-clamp electrophysiology and freshly isolated mouse brain capillary ECs, we examined whether Aβ_1–40_ or Tau-441 directly modulate Kir2.1 or Piezo1 function. Exogenously applied Aβ_1–40_ (10–100 nM) and Tau-441 (10–50 nM) had minimal effect on Kir2.1 current density, indicating that the Kir2.1 deficits observed in AD are less likely caused by direct interactions with Aβ or Tau. In contrast, we previously demonstrated that nanomolar Aβ_1–40_ enhanced Piezo1 function. We further show here that 50 nM Tau-441 significantly increased Piezo1 open probability, and this enhancement was abolished by the superoxide dismutase and catalase mimetic EUK-134. These data collectively suggest that altered Piezo1 function in neurodegenerative disease could involve a direct effect of Aβ peptides or Tau proteins and further suggest that acute exposure to these proteins minimally impacts Kir2.1 activity. These novel findings present a new pathway through which Tau proteins could impair neurovascular function during neurodegeneration.

## Significance

Tau and amyloid beta accumulation in the brain are hallmarks of Alzheimer’s disease, yet how they affect ion channels in the brain vasculature remains poorly understood. Using patch-clamp electrophysiology in freshly isolated mouse brain capillary endothelial cells, we show that nanomolar Tau-441 enhances the activity of the mechanosensitive channel Piezo1 in a reactive oxygen species-dependent manner, while leaving the potassium channel Kir2.1 unaffected. These findings identify a pathway through which Tau may impair on-demand cerebral blood flow.

## Introduction

Alzheimer’s disease (AD) is the most common cause of dementia and the most common neurodegenerative disease. AD pathogenesis is associated with the accumulation of amyloid-β (Aβ) peptides and Tau proteins in the brain.^1^ Aβ_1__–__40_ and Aβ_1__–__42_ are formed during improper splicing of the amyloid precursor protein, with Aβ_1__–__40_ being most common. Aβ peptides have been linked to cognitive decline and vascular dysfunction, including vascular inflammation and aging.^2^^,^^3^^,^^4^^,^^5^ On the other hand, Tau is a microtubule-associated protein that forms toxic oligomers in AD. While Tau plays an important role in stabilizing microtubules and regulating their assembly in the healthy brain, it is hyperphosphorylated in Tau-related diseases—known as tauopathies—leading to its dissociation from microtubule networks and oligomerization. These oligomers can move between neurons and vascular endothelial cells (ECs), where pathogenic Tau forms can further “recruit” wild-type Tau forms to dissociate from microtubules and oligomerize.^6^^,^^7^^,^^8^ Accumulation of human wild-type Tau is a hallmark of sporadic AD, with oligomers being proposed to be the primary toxic species. Tau oligomers associate with the brain vasculature in mouse models of AD and have been suggested to compromise cerebrovascular function.^9^

Brain capillary dysfunction has been recognized during AD pathology, reflecting the crucial role of capillaries for cerebrovascular health.^10^ Capillary endothelial cells (cECs) express several proteins in the healthy brain, including Kir2.1 and Piezo1 channels, that are critical in cerebral blood flow (CBF) regulation.^11^^,^^12^^,^^13^^,^^14^ The inwardly rectifying potassium channel Kir2.1 is important in controlling on-demand CBF increases, known as functional hyperemia.^11^ Kir2.1 senses neural activity due to the channel sensitivity to extracellular [K^+^] increases like those happening during neuronal firing. Sensing K^+^ activates Kir2.1 channels, hyperpolarizes cECs, generates a hyperpolarizing signal that dilates upstream arterioles, and ultimately increases local CBF. Importantly, reduced Kir2.1 activity—in mouse models of AD, hypertension, and cerebral small vessel diseases—is associated with CBF deficits.^15^^,^^16^^,^^17^^,^^18^^,^^19^ Piezo1, on the other hand, is a mechanosensitive nonselective cation channel that is also expressed throughout the brain endothelium.^13^ Mechanical activation of endothelial Piezo1 affects functional hyperemia by serving as a mechano-feedback mechanism that helps CBF recovery to baseline levels after hyperemia.^14^ Importantly, Piezo1 function in cECs is chronically elevated in a mouse model of familial AD.^20^ Despite the critical roles Kir2.1 and Piezo1 play in CBF regulation, whether Aβ peptides and Tau proteins affect their function remains poorly understood.

Here, we used patch-clamp electrophysiology to measure Kir2.1 and Piezo1 currents in brain cECs that are acutely exposed to exogenous Aβ or Tau. While we observed no effect for Aβ_1__–__40_ or Tau-441 on Kir2.1 currents, nanomolar Tau-441 increased Piezo1 activity in brain cECs, likely via reactive oxygen species (ROS) generation. These observations present a previously unknown pathway through which Tau proteins could impair CBF during neurodegeneration.

## Materials and methods

### Animals

Adult (3- to 5-month-old) male and female C57BL/6J mice (The Jackson Laboratory) were group housed on a 12-h light-dark cycle with environmental enrichment and free access to food and water. All experimental protocols used in this study are in accord with institutional guidelines approved by the Institutional Animal Care and Use Committee (IACUC) of the University of Vermont. This study complies with ARRIVE guidelines. We observed no differences between biological sexes in previous studies investigating Piezo1 and Kir2.1 channel activities^15^^,^^21^; both male and female mice were used in the current study.

### Isolation of brain cECs

Mice were euthanized with 5% isoflurane inhalation followed by decapitation. Brains were isolated and placed in an ice-cold physiological salt solution (5 mM KCl, 140 mM NaCl, 2 mM MgCl_2_, 4 mM glucose, and 10 mM HEPES). Tissues from the somatosensory cortex were homogenized with a Dounce homogenizer, filtered through a 62-μm nylon mesh, and then eluted in a dissociation solution (55 mM NaCl, 80 mM Na-glutamate, 5 mM KCl, 2 mM MgCl_2_, 10 mM glucose, and 10 mM HEPES [pH 7.3]) with neutral protease (0.5 mg/mL; Worthington Chemicals), elastase (0.5 mg/mL; Worthington), and 100 μM CaCl_2_. The tissues were then incubated at 37 C for 23 min. Collagenase type I (0.5 mg/mL; Worthington) was added for 2 min, and then the suspension was again filtered through 62-μm nylon mesh. The suspension was then triturated using a glass Pasteur pipette. A few drops of EC suspension were placed into a recording chamber containing enzyme-free dissociation solution and left for ∼30 min to adhere to the chamber. The cell suspension was kept on ice and used within ∼6 h. In all experiments, ECs were preincubated (30 min) with peptides/protein and/or drugs.

### Whole-cell patch-clamp electrophysiology

To measure Kir2.1 currents, cECs were bathed in bath solution (80 mM NaCl, 60 mM KCl, 1 mM MgCl_2_, 2 mM CaCl_2_, 4 mM glucose, 10 mM HEPES [pH 7.4]). Glass pipettes (Sutter Instruments) were pulled with a Narishige puller, fire polished to a tip resistance of 4–6 MΩ, and backfilled with pipette solution (10 mM NaOH, 11.4 mM KOH, 128.6 mM KCl, 1.1 mM MgCl_2_, 2.2 mM CaCl_2_, 5 mM EGTA, 10 mM HEPES [pH 7.2]). Whole-cell currents were recorded using a patch-clamp amplifier (Axopatch 200B; Molecular Devices) and digitizer (Digidata 1550B; Molecular Devices). Recordings were filtered at 1 kHz, digitized at 5 kHz, and stored for subsequent analysis using Clampfit 11.3 (Molecular Devices). A sampling rate of 10 kHz and a low-pass filter frequency of 1 kHz were used. Whole-cell capacitance was measured using the cancellation circuitry in the voltage-clamp amplifier. Currents were recorded within 3–5 min of gaining electrical access to the interior of the cell to minimize the potential impact of current rundown.

### Cell-attached Piezo1 electrophysiology

Single-channel Piezo1 currents were recorded in the cell-attached, voltage-clamp configuration as described previously.^20^ Cells were placed in bath solution (140 mM KCl, 1 mM MgCl_2_, 4 mM glucose, 2 mM CaCl_2_, 10 mM HEPES [pH 7.35]). Borosilicate glass pipettes were fire polished to a tip resistance of ∼5 MΩ and filled with pipette solution (3 mM KCl, 137 mM NaCl, 1 mM MgCl_2_, 2 mM CaCl_2_, 4 mM glucose, 10 mM HEPES [pH 7.35]). The pipette solution was supplemented with 5 μM Yoda1 (pharmacological Piezo1 activator). Recordings were made at −50 mV after achieving a giga-ohm seal. Minimum accepted recording duration was 300 s. Open probability (NP_O_), mean open time, and mean closed time were calculated by threshold-crossing analysis.

### Peptide preparation and application

Lyophilized native Aβ_1–40_ (A-1157; rPeptide) and scrambled Aβ_1–40_ (A-1003; rPeptide) were resuspended in 1% NH_4_OH and diluted with phosphate-buffered saline to 0.1–1 mg/mL. The solution was sonicated for 5 min, aliquoted, and stored at −20°C. Scrambled Aβ_1–40_ served as a negative control. Aβ_1–40_ was applied onto ECs by supplementing the cell suspension solution (30-min preincubation), the bath solution, and the pipette solution with a final concentration of 10 nM or 100 nM. Solutions were sonicated for ≥5 min before experimentation. Lyophilized Tau-441 (T-1001; rPeptide) was resuspended in water to 0.1–1 mg/mL, sonicated for 5 min, aliquoted, and stored at −20°C. Tau-441 was applied identically to Aβ at final concentrations of 10 nM or 50 nM. In ROS inhibition experiments, EUK-134 (10 μM; Cayman Chemical) was co-applied with Tau-441. GSK2193874 (Tocris Bioscience) and barium chloride (Sigma) were included in the pipette to block TRPV4 and Kir2.1 currents.

#### Statistics and analyses

Statistical analysis was performed using GraphPad Prism version 10. Kir2.1 current density data were analyzed by one-way ANOVA with Dunnett’s test for multiple comparisons against the no-peptide control. Piezo1 NP_O_, open time, and closed time data were analyzed by one-way ANOVA with Dunnett’s multiple comparisons test. Data are presented as mean values ±SEM. *p* < 0.05 was considered significant. Sample sizes are reported as *n* = ECs/mice.

## Results

### Aβ_1__–__40_ and Tau-441 do not affect Kir2.1 current density in brain capillary ECs

**Figure 1:**
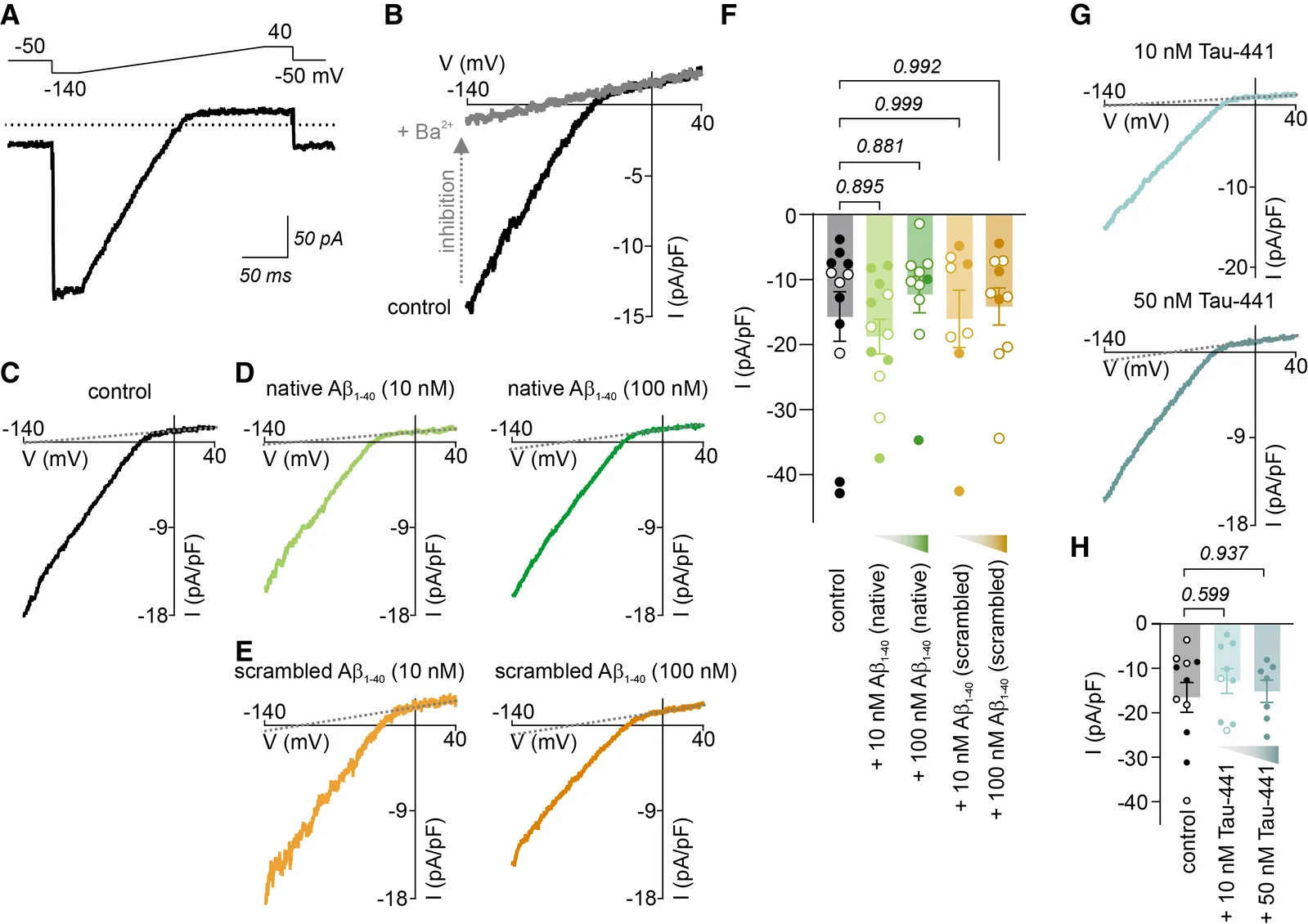
Kir2.1 channel function is unchanged by Aβ_1__–__40_ or Tau-441. (A) Voltage ramp protocol (−140 to +40 mV from −50 mV holding potential) and representative recorded Kir2.1 currents without peptide. (B) Example I-V relationship showing Kir2.1 current inhibition by 100 μM Ba^2+^ compared with control. Ba^2+^ was only to confirm Kir2.1 channel identity through its known blocking effect, not to quantify Kir2.1 current density, which was determined from baseline recordings in its absence. (C) Representative Kir2.1 control current recorded in the absence of peptide. Recording configuration: pipette solution contained 140 mM K^+^; bath solution contained 60 mM K^+^. The dotted line represents the extrapolated linear (ohmic) current expected in the absence of inward rectification, illustrating the deviation characteristic of Kir2.1 channel behavior. (D) Kir2.1 currents in the presence of 10 nM (left) and 100 nM (right) native Aβ_1-40_. (E) Example currents in the presence of 10 nM (left) and 100 nM (right) scrambled Aβ_1-40_. (F) Scatterplot of Kir2.1 current density (pA/pF) across all Aβ conditions (no peptide, *n* = 12 ECs/6 mice [3 female mice]; 10 nM Aβ_1-40_ native, *n* = 12/4 [2F]; 100 nM Aβ_1-40_ native, *n* = 10/4 [3F]; 10 nM Aβ_1-40_ scrambled, *n* = 8/3 [1F]; 100 nM Aβ_1-40_ scrambled, *n* = 10/4 [2F]). Open symbols represent ECs from female mice. (G) Representative Kir2.1 currents in the presence of 10 nM (top) and 50 nM (bottom) Tau-441. (H) Scatterplot of Kir2.1 current density for Tau-441 conditions (no peptide, *n* = 11/4 [2F]; 10 nM Tau-441, *n* = 8/3 [1F]; 50 nM Tau-441, *n* = 7/2 [0F]). All peptides were present in both bath and pipette solutions. Data in (F) and (H) are presented as mean ± SEM. All comparisons were made using the one-way ANOVA test and Dunnett’s test for multiple comparisons, *p* values are included.

Kir2.1 is required for functional hyperemia,^11^^,^^12^ and its activity is impaired in models of AD.^15^^,^^16^^,^^17^ Whether Aβ_1–40_ or Tau-441 directly modulate Kir2.1 function in brain capillary ECs has not been tested. Using conventional whole-cell patch-clamp electrophysiology, we measured Kir2.1 current density in freshly isolated mouse brain capillary ECs exposed to Aβ_1–40_ or Tau-441. Whole-cell currents were evoked by voltage ramps from −140 to +50 mV (from a holding potential of −50 mV) in a high-potassium (60 mM K^+^) bath solution, used to increase Kir2.1 current amplitude (Figure 1A). Current density was obtained by dividing peak inward current (at −140 mV) by whole-cell capacitance, and currents were sensitive to Ba^2+^ (100 μM) application to the bath solution (Figure 1B). A low-nanomolar concentration of Aβ_1–40_ is sufficient to evoke cerebrovascular dysfunction.^22^ We therefore first recorded Kir2.1 current density in the presence of 10 nM native or scrambled Aβ_1–40_. No significant difference was detected among ECs recorded without peptide, with 10 nM scrambled Aβ_1–40_, or with 10 nM native Aβ_1–40_. At a higher concentration of 100 nM, neither scrambled nor native Aβ_1–40_ affected Kir2.1 currents compared with no-peptide control (Figures 1C–1E). These data suggest that exogenous Aβ_1–40_ does not acutely alter Kir2.1 channel activity in brain capillary ECs (Figure 1F). Extracellular, nonphosphorylated Tau contributes to synaptic dysfunction in AD,^18^ and recombinant full-length Tau applied to mouse brains is associated with cerebrovascular dysfunction.^7^^,^^23^ To test whether this effect could involve Kir2.1 modulation, we recorded whole-cell currents in the presence of 10 nM and 50 nM Tau-441. At either concentration, Kir2.1 current density was indistinguishable from untreated cells (Figures 1G and 1H). These observations collectively suggest that the Kir2.1 deficits seen in AD cannot be not explained by acute interactions with soluble Aβ_1–40_ or Tau-441.

### Tau-441 increases Piezo1 open probability in brain capillary ECs

**Figure 2:**
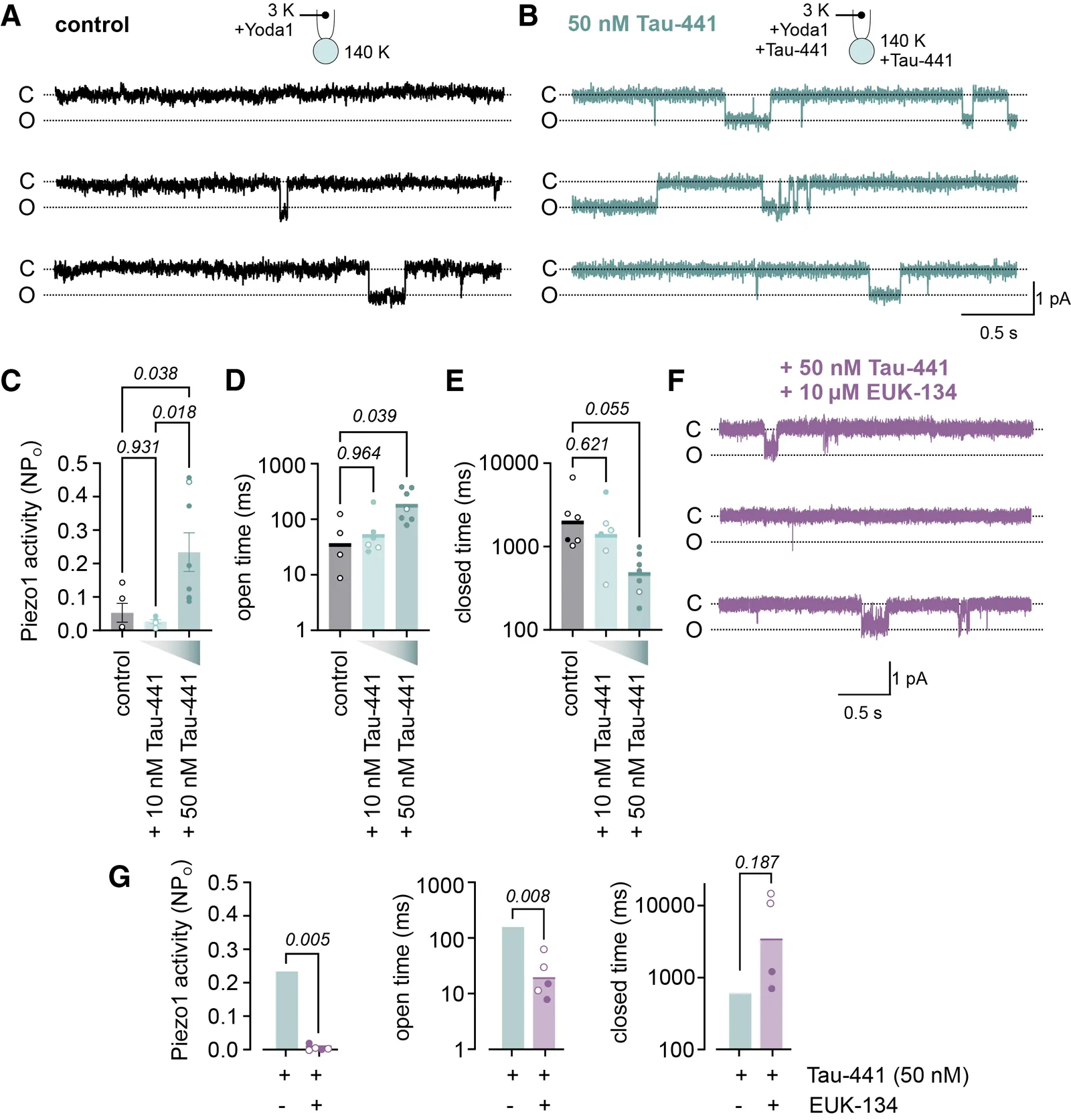
Tau-441 enhances Piezo1 activity. (A) Representative cell-attached single-channel traces under control conditions (no peptide). Currents were measured in the presence of Yoda1 (5 μM), and patches were held at −50 mV. C denotes closed; O denotes one channel open. (B) Representative single-channel traces in the presence of 50 nM Tau-441. (C) Scatterplots of Piezo1 NP_O_ under control, 10 nM, and 50 nM Tau-441 conditions (no peptide, *n* = 5/3 “ECs/mice” [0 female mice]; 10 nM Tau-441, *n* = 5/2 [1F]; 50 nM Tau-441, *n* = 8/5 [1F]). (D) Scatterplots of mean channel open time across the same conditions (*n* = 4–7 ECs from 7 mice [3F]). (E) Scatterplots of mean channel closed time across the same conditions (*n* = 6–7/6 [3F]) (F) Representative traces in the presence of 50 nM Tau-441 + 10 μM EUK-134. (G) Scatterplots of Piezo1 NP_O_, open time and closed times comparing 50 nM Tau-441 alone (*n* = 8/5) versus 50 nM Tau-441 + 10 μM EUK-134 (*n* = 4–5/3 [2F]). All comparisons were made using the one-way ANOVA test and Tukey’s test for multiple comparisons, *p* values are included.

Piezo1 is expressed in brain capillary ECs,^13^^,^^14^ and its activity is enhanced by Aβ_1–40_.^21^ Whether Tau-441 affects Piezo1 function has not been explored. We measured single-channel Piezo1 activity in the cell-attached configuration in freshly isolated mouse brain capillary ECs. Cells were preincubated with Tau-441 for 30 min, with Tau-441 present in both the bath and pipette solutions during recordings (Figures 2A and 2B). Baseline Piezo1 NP_O_ in the presence of the pharmacological Piezo1 activator Yoda1 (5 μM) and absence of Tau proteins was 0.05 ± 0.03. Application of 10 nM Tau-441 did not significantly alter NP_O_ (Figure 2C), but 50 nM Tau-441 produced a significant ∼4-fold increase in Piezo1 function compared with control (Figures 2B and 2C). This NP_O_ increase was accompanied by longer channel open times and a trending reduction in closed times (Figures 2D and 2E). These data demonstrate that exogenous Tau-441 enhances Piezo1 activity.

### Tau-441-enhanced Piezo1 activity is abolished by ROS inhibition

Tau accumulation is associated with increased ROS production in tauopathies,^24^^,^^25^^,^^26^ and ROS have been shown to enhance endothelial Piezo1 activity in response to amyloid peptides.^21^ We therefore asked whether the Tau-441-mediated increase in Piezo1 NP_O_ is sensitive to ROS suppression. To test this, the superoxide dismutase and catalase mimetic EUK-134 (10 μM) was co-applied with Tau-441 onto capillary ECs (Figures 2F and 2G). In the presence of 10 nM Tau-441, where no significant change in NP_O_ was detected, the co-application of EUK-134 did not produce a further change. Strikingly, the significant increase in Piezo1 NP_O_ evoked by 50 nM Tau-441 was completely abolished by EUK-134 co-treatment. Mean open time was similarly restored, and closed time showed trending increases toward control levels when EUK-134 was used (Figures 2F and 2G). These results suggest that Tau-441 enhances Piezo1 activity through a ROS-dependent mechanism.

## Discussion

This study provides evidence that Tau protein enhances Piezo1 channel activity in brain capillary ECs and further shows that this enhancement could be reversed by blocking ROS generation. These findings are in line with previous findings from our group that Aβ_1–40_ enhanced Piezo1 currents in brain capillaries.^21^ In distinction, neither Aβ_1–40_ nor Tau-441, at concentrations sufficient to cause cerebrovascular dysfunction *in vivo*, acutely altered Kir2.1 current density. Together, these findings suggest that direct interactions with soluble, full-length Aβ_1–40_ or Tau-441 do not underlie the Kir2.1 deficits seen in AD models, while Tau-441 acts—presumably via a ROS-dependent pathway—to facilitate Piezo1-mediated cation influx.

Kir2.1 is essential for functional hyperemia,^11^^,^^12^ and its impairment is a consistent feature of AD models.^15^^,^^17^^,^^22^ It was previously hypothesized that AD and Aβ peptides impair Kir2.1 through compromising the levels of the phosphoinositide PIP_2_ (phosphatidylinositol-4,5-bisphosphate) or reduced channel expression.^15^^,^^27^ Our data argue against a direct, acute mechanism: Kir2.1 current density was unchanged across a 10-fold concentration range of Aβ_1–40_ (10–100 nM) and by Tau-441 (10–50 nM). The Kir2.1 deficits seen in AD could therefore reflect indirect mechanisms that accumulate over time—such as chronic oxidative stress, altered channel trafficking, or PIP_2_ depletion—rather than channel-peptide interactions. Consistent with this possibility, Kir2.1 function is selectively impaired by long-term but not short-term oxidative stress,^28^ suggesting that a prolonged exposure paradigm could better recapitulate the *in vivo* deficits.

To the best of our knowledge, interactions between Tau and endothelial Piezo1 had not been examined. It is worth noting that a recent study demonstrated that the cytotoxic T-peptide, derived from microtubule binding repeat of Tau protein, disrupted endothelial Ca^2+^ signaling and cerebrovascular vasodilation.^29^ Importantly, the T-peptide used in the previous study^29^ is short (15 amino acids), low molecular weight (2.2 kDa) Tau-derived peptide that is cytotoxic across multiple cell types.^30^ This is quite distinct from the full-length, mature human Tau isoform with all 441 amino acids (i.e., ∼46 kDa) that was tested here. Notably, we used monomeric, nonphosphorylated Tau rather than the hyperphosphorylated oligomers and fibrils that predominate in AD. Wild-type Tau oligomers have been proposed as the primary toxic species in sporadic AD, and exploring oligomeric preparations may reveal larger or qualitatively distinct effects.^31^^,^^32^ Pharmacological scavenging of ROS completely abolished the effect of Tau-441, implicating ROS as the potential mechanistic link between Tau exposure and Piezo1 activation. This finding parallels our previous demonstration that Aβ_1–40_ activates Piezo1 through a ROS-dependent mechanism as evidenced by sensitivity to superoxide dismutase/catalase mimetics and NADPH oxidase inhibition.^21^ Future studies examining the combined effects of Aβ and Tau on capillary ion channels will be important to fully define how AD-associated peptides alter brain capillary electrophysiology. Together, our findings suggest that ROS-mediated Piezo1 activation may be a convergent pathway by which multiple AD-associated peptides damage brain capillary function.

While Tau-induced mitochondrial dysfunction and NADPH oxidase activation have been proposed as sources of ROS, the precise mechanisms remain incompletely understood and warrant further investigation. Tau hyperphosphorylation and oxidative stress are closely linked in tauopathies,^24^ and one proposed mechanism involves Tau-induced upregulation of mitofusins, leading to mitochondrial dysfunction and secondary ROS generation.^33^ Further, ROS-induced lipid peroxidation increases plasma membrane tension, lowering the activation threshold of mechanosensitive channels such as Piezo1.^34^ Sustained Piezo1 activation would be expected to increase cation influx and depolarize capillary ECs, suppressing the Kir2.1-dependent signaling that drives functional hyperemia.^11^^,^^14^ Whether Tau-induced Piezo1 activation contributes to the neurovascular dysfunction observed in tauopathy mouse models^35^ remains an important question for future study.

Overall, this study identifies Tau-441 as a modulator of Piezo1 activity in brain capillaries and implicates ROS generation as an underlying mechanism. These findings extend our understanding of how Tau pathology may compromise CBF regulation and position the Piezo1-ROS axis as a candidate target for therapeutic intervention in neurodegenerative disease.

## Data availability

All data are presented in the main manuscript.

## Acknowledgments

The authors thank Drs. Xin Rui Lim, Megan Tuineau, and Mohammad Elmahdy for technical assistance and valuable discussions. This work was supported by a Summer Research Award from the 10.13039/100010941University of Vermont Biochemistry Department (T.H.). Research in the Harraz Laboratory was supported by the 10.13039/100000050National Heart, Lung, and Blood Institute (R01-HL169681), the 10.13039/100000049National Institute on Aging (R21-AG082193), the 10.13039/100000057National Institute of General Medical Sciences (P20-GM135007), the 10.13039/100000968American Heart Association (20CDA35310097), the 10.13039/100014989Chan Zuckerberg Initiative (2024-338506), the cureCADASIL Association (52018063), the Bloomfield Early Career Professorship in Cardiovascular Research, the Totman Medical Research Trust, the 10.13039/100018583Larner College of Medicine at the University of Vermont, and the 10.13039/100020397Cardiovascular Research Institute of Vermont.

## Author contributions

Conceptualization, O.F.H.; investigation and analysis, T.H. and O.F.H.; writing, T.H. and O.F.H.; funding acquisition, T.H. and O.F.H.; project administration, O.F.H.

## Declaration of interests

The authors declare no competing interests.
